# Substitutional landscape of a split fluorescent protein fragment using high-density peptide microarrays

**DOI:** 10.1371/journal.pone.0241461

**Published:** 2021-02-03

**Authors:** Oana N. Antonescu, Andreas Rasmussen, Nicole A. M. Damm, Ditte F. Heidemann, Roman Popov, Alexander Nesterov-Mueller, Kristoffer E. Johansson, Jakob R. Winther

**Affiliations:** 1 Department of Biology, Linderstrøm-Lang Centre for Protein Science, Section for Biomolecular Sciences, University for Copenhagen, Copenhagen, Denmark; 2 Institute of Microstructure Technology, Karlsruhe Institute of Technology, Eggenstein-Leopoldshafen, Germany; Panjab University Chandigarh, UNITED STATES

## Abstract

Split fluorescent proteins have wide applicability as biosensors for protein-protein interactions, genetically encoded tags for protein detection and localization, as well as fusion partners in super-resolution microscopy. We have here established and validated a novel platform for functional analysis of leave-one-out split fluorescent proteins (LOO-FPs) in high throughput and with rapid turnover. We have screened more than 12,000 variants of the beta-strand split fragment using high-density peptide microarrays for binding and functional complementation in Green Fluorescent Protein. We studied the effect of peptide length and the effect of different linkers to the solid support. We further mapped the effect of all possible amino acid substitutions on each position as well as in the context of some single and double amino acid substitutions. As all peptides were tested in 12 duplicates, the analysis rests on a firm statistical basis allowing for confirmation of the robustness and precision of the method. Based on experiments in solution, we conclude that under the given conditions, the signal intensity on the peptide microarray faithfully reflects the binding affinity between the split fragments. With this, we are able to identify a peptide with 9-fold higher affinity than the starting peptide.

## Introduction

Tens of new split fluorescent proteins (splitFPs) have been developed since the first reassembly of a splitFP was achieved 20 years ago [1]. Fluorescent proteins (FPs) have been split in many creative ways, by removing fragments ranging from around half of the FP β-barrel [1] to one [2] or two [3] secondary elements. The splitFP fragments obtained have low or no fluorescence on their own, but can reassemble to form a fully functional FP. These properties have made splitFPs desirable for many bioanalytical applications, from sensing protein-protein interactions to protein detection and localization, and as tools in super-resolution microscopy [4,5].

Leave-one-out splitFPs (LOO-FPs) are variants of splitFPs in which one of the secondary elements, such as one of the β-strands or the internal α-helix, typically of less than 20 amino acids, are removed [6]. Ideally, left-out elements can spontaneously associate with the LOO-FP to recover fluorescence, making them useful as tags fused to a protein of interest, as well as individual peptides for *in vitro* applications. Preferably, a peptide should have high solubility, affinity and brightness upon complementation of the LOO fragment, as well as a small size that interferes minimally with a potential fusion partner protein [2,5,7,8]. Amino-acid substitutions in the sequence of the LOO-FP fragments offer the possibility to modulate their complementation efficiency, spectral properties, solubility and photostability. Although these are clear targets for optimizing LOO-FPs, genetic engineering methods such as random mutagenesis are laborious and do not directly measure binding between the splitFP fragments [9].

High-density peptide microarrays provide a powerful technology for massively parallel screening of peptide-protein interactions. While DNA and RNA arrays have been extensively used in mappings of polynucleotide-protein interactions [10], peptide microarrays have mostly been reserved for antibody epitope mapping and screening receptor-ligand interactions [11]. Peptide microarrays cannot, however, typically accommodate peptides longer than 15–20 residues and, in addition, the affinity of the peptide-protein interaction to be investigated needs to be less than μM [11]. Thus, peptide microarray analysis is attractive for complementing LOO-FPs, as peptides are generally under 20 residues [6], and can bind the LOO partner with dissociation constants from hundreds of picomolar to hundreds of nanomolar [4].

LOO-FPs systems can be divided in those where the chromophore is matured prior to reconstitution of the full-length protein and those were maturation takes place on reconstitution. While fluorescence recovery in the former is rapid (in the order of minutes) and essentially dependent on the rate of association of the partners, the latter may take hours requiring chemical condensation and oxidation of the chromophore. We hypothesized that, since binding of the split fragments with a preformed chromophore immediately generates a fluorescent signal, their association could rapidly be followed by fluorescence detection on a peptide microarray. Thus, we opted for a system in which the chromophore was matured prior to reconstitution, namely the *superfolder* “GFP split10” system, in which strand 10 is removed from the N-terminus of a circular permutated variant by trypsin digestion generating LOO10-GFP [12]. The peptide microarray that we tested had a library of 12,544 left-out strand 10 (s10) peptide sequences in 12-fold repeats and was screened for complementation to the partner truncated protein using a fluorescence laser scanner. Contrary to DNA-based screening methods, chemical synthesis of defined peptides allowed for highly targeted analysis in which specific sequences can be queried in a non-random fashion with direct, rapid and quantitative readout.

We generated comprehensive splitFP sequence-function maps in a single experiment and with high precision, without the need of mutant selection rounds, enrichments or individual handling of clones. Analyzing peptides scanned with all possible amino acid substitutions in s10, we mapped hotspot residues and discovered improving substitutions. SplitFP complementation using peptide arrays also provided information about the sequences with lower fluorescence yield, which are generally inaccessible by genetic methods [2,7]. By introducing variations in the s10 context such as the peptide length and charge of the C-terminal array surface linker, we demonstrated the robustness of the assay. Finally, we assessed the accuracy of the microarray platform by characterizing interesting s10 sequences spectrally and thermodynamically in solution.

## Results

### Experimental setup

**Fig 1:**
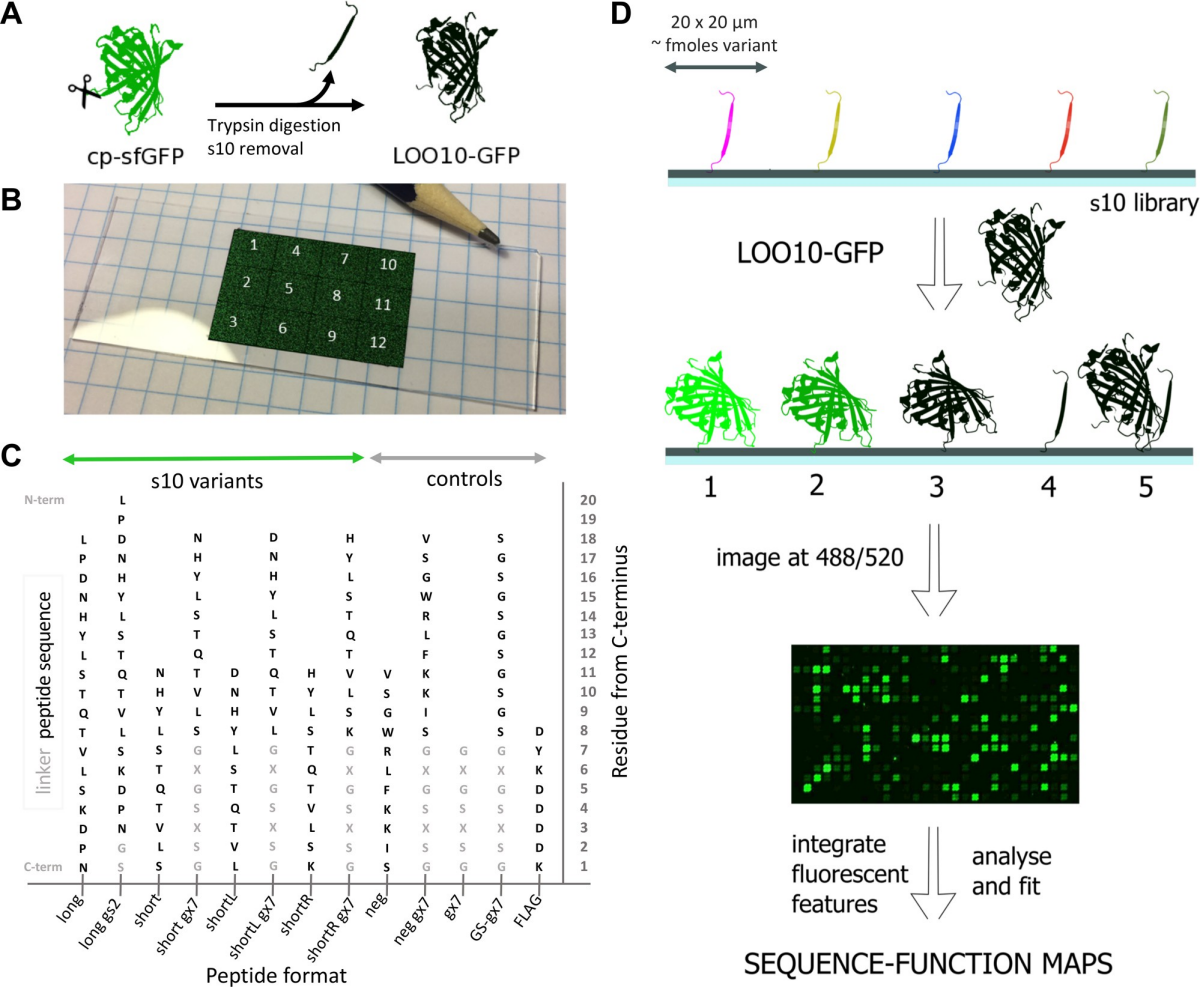
Experimental setup. (A) LOO10-GFP is obtained by removing strand 10 from a circularly permuted superfolder GFP; (B) Peptide microarray layout with 12 identical sectors (pencil tip for size appreciation). In each sector, peptide variants are bound to the solid surface at the C-terminus, forming well defined rectangular 20 x 20 μm spots—here pasted onto the slide in false color for visualization. (C) The peptide formats present in the library are long and short length variants with or without a gs2 or gx7 linker. Controls are also synthesized with or without linker. Linker sequences tested are gs2 –GS and gx7, which can be gs7 –GSGSGSG, gk7 –GKGSKSG or ge7 –GEGSESG. S10 and control sequences are colored black, while the linker sequences light gray. Substitutions were only performed in the s10 sequences and not in the linkers. (D) The peptide microarray is incubated with LOO10-GFP and imaged in the green channel. Scenarios at the microarray surface upon incubation: 1. Specific binding and high fluorescence; 2. Specific binding and low fluorescence; 3. Specific binding and no fluorescence; 4. No binding; 5. Non-specific binding. The fluorescence images were registered to the sequence information, then integrated and fit to sequence-function maps.

LOO10-GFP was obtained from a *circularly permuted superfolder* GFP (cp-sfGFP), with β-strand 10 engineered in the N-terminal, employing minor modifications to an established protocol [12]. S10 was removed by trypsin digestion followed by size exclusion chromatography in denaturing conditions, to yield LOO10-GFP (Fig 1A). Upon refolding in native buffer, LOO10-GFP was obtained in a fairly stable and soluble state which recovered fluorescence when reassembled with synthetic s10 variants in solution (S1 Fig).

The s10 peptide library was designed as a single microarray layout with 12 identical sectors, amounting to 150,528 peptide spots in total on a microscope-format slide (illustrated in the false-color image Fig 1B). The s10 library is developed around the 18-mer “wild-type” s10 peptide L_195_PDNHYLSTQTVLSKDPN_212_ (termed s10_long_). Preliminary experiments had shown that a truncation to a core sequence of 11 residues, N_198_HYLSTQTVLS_208_ (s10_short_), forming a minimal β-strand, was also able to complement in solution (S1 Fig). In addition, preliminary microarray screens showed that 7-mer charged linkers for the short format would be beneficial (S2 Fig). Thus, 7-mer linkers with positive, neutral and negative charges were screened for s10_short_ in order to increase signal to noise, but also to assess signal robustness. We also wished to study how 1 amino acid shifts to the left and right of the sequence of s10_short_ influenced fluorescence recovery, resulting in the s10_shortL_ D197-L207 and the s10_shortR_ H199-K209 variants, respectively. An overview of the length, linker and substitution variants tested for the s10 peptide can be found in Fig 1C and S1 Table. To generate a comprehensive picture of the sequence tolerance, all single amino acid substitutions were synthesized for the s10_long_ (342 variants), s10_short_ (209 variants), s10_shortL_ (209 variants) and s10_shortR_ (209 variants) formats. In addition, a series of double (1607 variants for s10_long_, 942 variants for s10_short_) and triple substitutions (304 variants for s10_long_, 171 variants for s10_short_) were added at positions 199, 203 and 207. Particular substitutions at positions 199 and 203 had in preliminary screens shown enhanced fluorescence (S2 Fig), while substitutions at positions 207, were included in libraries as negative controls. To access the effect of linkers, all the single, double and triple substitutions were assessed both without linker and linked to GS (*gs2*) for s10_long_ and GSGSGSG (*gs7*), GKGSKSG (*gk7*) and GEGSESG (*ge7*) for s10_short_. As controls, substitutional scans of a negative control (*neg*) 11-mer peptide inspired from a split-luciferase [13] were synthesized to test the specificity of LOO10-GFP to s10 variants. Finally, we included linker and FLAG synthesis controls, and blank spots for background estimation.

Arrays were synthesized on modified surface microscope slides using a lithographic method described previously [14] and were purchased from a commercial vendor (Schafer-N, Copenhagen). Identical peptides were placed at the same position relative to other peptides within individual sectors, but peptides were deliberately *not* grouped by linker or composition within a sector, but rather placed randomly to avoid local artifacts affecting similar peptides in a similar fashion.

To determine binding and recovery of activity, we incubated the peptide microarray with LOO10-GFP, and after a 10 min wash step the microarray was briefly dried and imaged at 488 nm excitation and 520 nm emission using a microarray scanner (see Methods for details; Fig 1D). Image analysis of the fluorescent spots allowed us to assign each peptide in the library a fluorescent signal.

### Microarray assay performance

Before determining any substitutional effects, we quantitatively assessed the performance of the microarray assay in terms of precision, specificity and robustness. Data handling and statistical analyses are detailed in Methods. The high precision of the microarray method was proven by correlating the variant libraries in all 12 sector replicas, which showed Pearson coefficients > 0.94 (S3 Fig).

**Fig 2:**
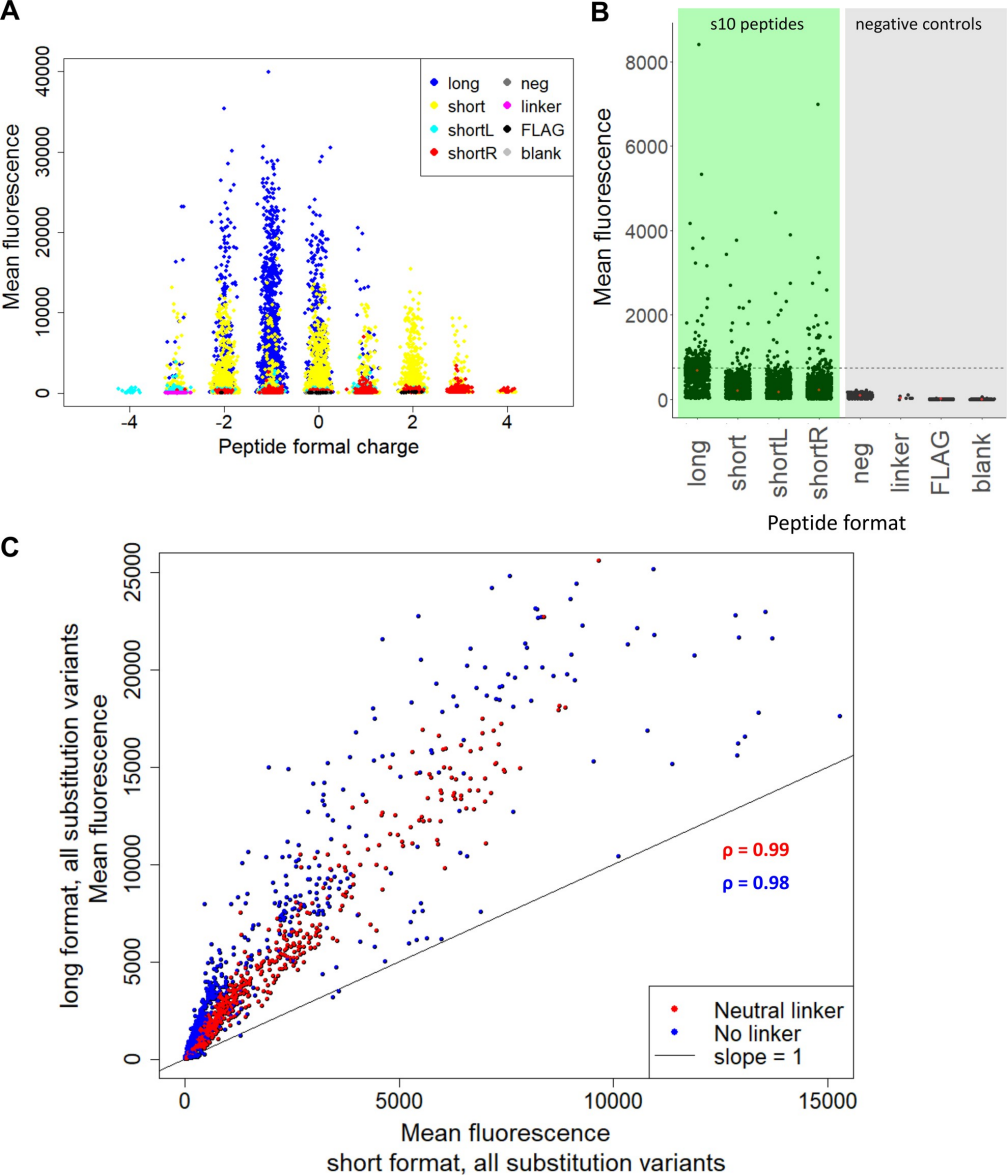
Microarray assay performance. (A) Mean fluorescence against formal charge of each peptide variant. The charge was estimated by counting all positive (R, K) and negative (D, E) charges in each sequence. (B) Mean fluorescence of linker and substitution variants for the long, short, shortL and shortR s10 peptides (green background), compared to neg, linker, FLAG and blank controls (grey background). The dashed line shows the fluorescence of s10long WT, that we used as positive control in this dataset. Double and triple substitutions were excluded, since they were only performed on the long and short formats. (C) Red points indicate mean fluorescence of all variants with neutral linker in the short format versus the same variants of long format. Blue points indicate mean fluorescence of all substitution variants without linker of the short format versus the same variants of long format. The indicated *ρ* values are Pearson coefficients of the two correlations.

Binding to positively charged peptides by low pI proteins is a common concern with peptide microarrays [15,16]. We tested whether the low-fluorescence LOO10-GFP might have non-specific bias towards highly charged peptide spots, leading to false-positive signals. Results showed that microarray fluorescence was independent of formal peptide charges and distributes around s10_long_ WT, which has a formal net charge of -1 (Fig 2A). In addition, the signals from negative control peptides were minimal compared to the fluorescence from s10 peptide variants, suggesting specificity. (Fig 2B). Comparing long and short length variants we found that peptide length and charged C-terminal linker variants can have an influence on the dynamic range of fluorescence. In Fig 2C we compared the same substitution variants in the long versus the short peptide format, with or without a neutral linker. The long and short variants correlated well with each other (Pearson coefficients > 0.98), but the signal from s10_long_ peptides was substantially higher than from s10_short_ format in terms of the dynamic range.

The various linkers of the short formats are all well correlated for individual substitutions among themselves. For the s10_short_ format a linker is preferable, presumably to increase the distance to the microarray surface. This is seen from the increased scatter of the data comparing linker-free versus linker-containing peptides (Pearson coefficients 0.92–0.93) as opposed to the much higher correlation between linker containing peptides (Pearson coefficients 0.99; S4 Fig). Using the gk7 or ge7 linkers with formal charge +2 or -2 respectively appears to result in slightly higher signal as compared to the neutral gs7 linker (S4 Fig). The shifted variants had low signal-to-noise, but seem to follow a nearly one-to-one relationship with short variants (S5 Fig). This suggests that the 9-mer central sequence HYLSTQTVL could potentially work *per se* as a split 10 fragment.

The main conclusion from this comparison is that although there are differences in the performance of individual linker designs, the variant sequences correlated very well with each other between linker formats. Overall, the microarray signals are highly robust with regards to ranking across substitution variants. However, due to the different dynamic ranges obtained at different length and linker formats, substitutional effects should always be interpreted within the same peptide format.

### Effect of substitutions on s10long array signal

**Fig 3:**
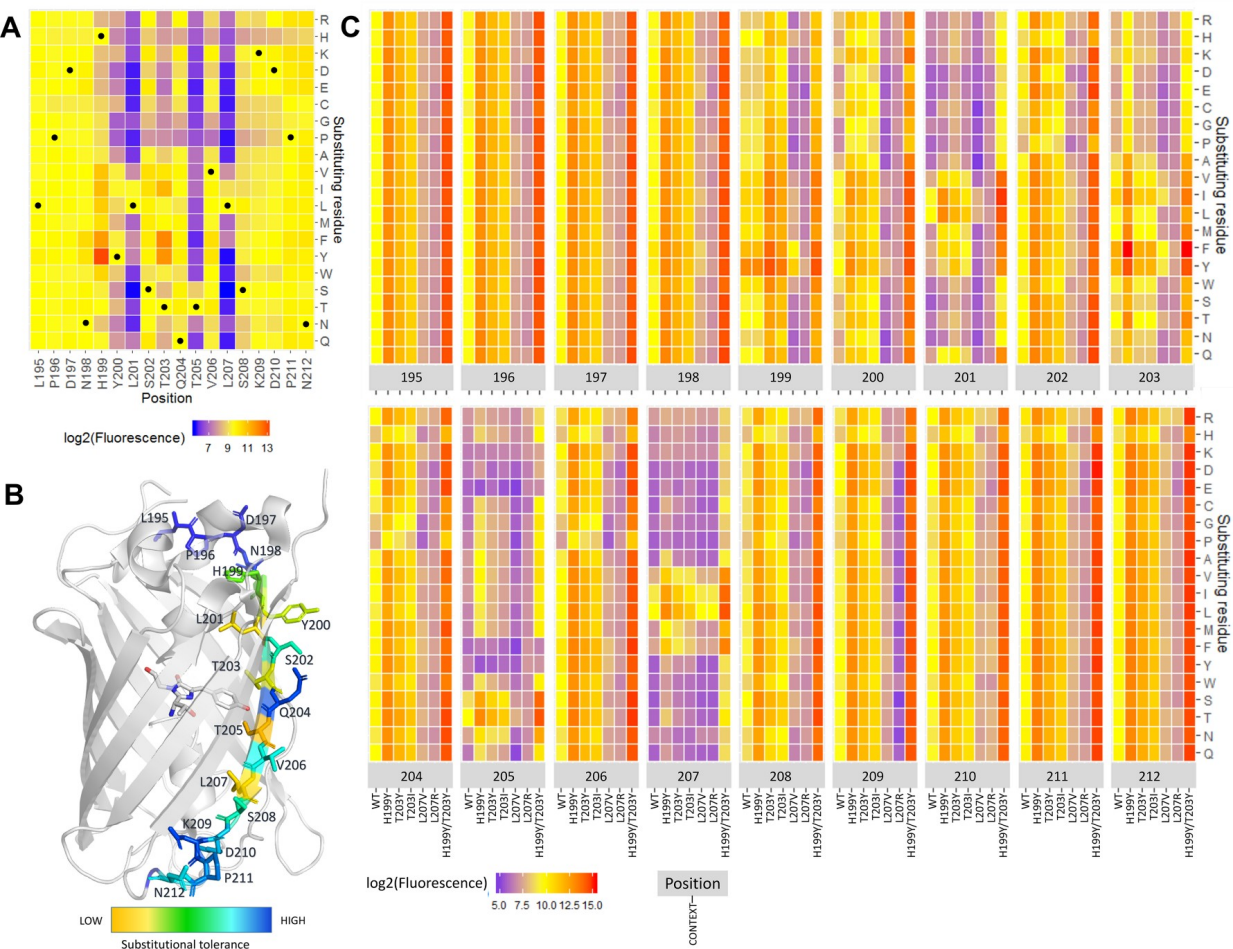
Effect of substitutions on s10long fluorescent signal. (A) Saturation substitution heatmap of the s10long peptide. In heatmaps log2(Fluorescence) is used; Yellow–WT-like fluorescence; Blue–loss-of-function substitutions; Red–gain-of-function substitutions (for details, see Methods section). (B) Substitutional tolerance values from 1 (low) to 19 (high) are color-scaled on the superfolder GFP structure (PDB: 2B3P) with the s10 side chains and chromophore represented as sticks. (C) Fluorescence heatmap of single, double and triple substitutions from s10 WT, where log2(Fluorescence) is shown for substitution variants (y-axis) at each s10 position (higher x-axis) in the WT, H199Y, T203Y, T203I, L207V, L207R and H199Y/L203Y context (lower x-axis). Median relative standard deviation RSD% across all variants was 15%.

Since all peptide formats gave sequence-dependent correlated signals, we will in the following discuss substitutional effects in the s10_long_ format without linker, since this format yielded the highest signals and dynamic range. The heatmap in Fig 3A shows the effect of all s10_long_ single residue substitutions on the fluorescence of the s10_long_:LOO10-GFP complex. Some increase in fluorescence when substituting T203 with hydrophobic residues Y, F, I and V was expected, because these substitutions are known to cause a red shift in the fluorescence emission maximum from 506 nm to, depending on the context, 515–527 nm [12,17–19]. The readout using a 520 nm emission filter could favor the T203 red-shifted substitutions. Besides the effects at position 203, replacing H199 with a series of hydrophobic residues (Y, F, I, V, L) also resulted in increased signal. Specifically, introducing H199Y as a single substitution offered 10-fold greater fluorescence compared to WT.

To evaluate the importance of each of the WT residues, we assigned each s10 position a substitutional tolerance value from 1 (lowest) to 19 (highest) based on the number of substitutions it could accept at each position without gaining or losing function, as described in Methods. Mapping this onto the superfolder GFP crystal structure (Fig 3B) showed that positions with lowest substitutional tolerance were 199, 200, 201, 203, 205, 207, all pointing inside the FP β-barrel except position 200. The low tolerance positions delimit the 199–207 region as the s10 core residues for function. Within core region, changing outward-pointing residues 202, 204, 206 to β-sheet-disruptive P and G were, as expected, detrimental [20]. We noted that substitution to H had a negative impact on almost all positions.

In the peptide library we included full substitutional scans of s10_long_ H199Y, T203Y, T203I, L207V, L207R and H199Y/T203Y peptides, these libraries representing double and triple substitutions from WT. A full overview of the functional recovery of all tested single, double and triple substitutions from s10 WT is shown in the heatmap in Fig 3C. Most of the substitutional variants in the H199Y, T203Y and T203I contexts were distributed at fluorescence values above WT, increasing the fluorescence on average by a factor of 9.1, 4.2 and 2.4, respectively, but there were also many with less signal than WT (S6 Fig). In the H199Y/T203Y context, which increased the fluorescence on average by a factor of 23, almost all substitutions are gain-of-function, with some exceptions for substitutions in positions 205 and 207. For example, L207V is rescued at 3-fold WT levels in presence of H199Y. On the other hand, all substitutions in the L207R context are loss of function, meaning that there is no substitution that can rescue L207R. Only substitutions in positions 201, 205 and 207 can bring the fluorescence of variants in the high-performing H199Y/203Y context below WT levels (S6 Fig), suggesting that these are the three most constrained s10 positions.

H199Y was the most fluorescence enhancing single substitution and the most robust when combined with other substitutions, obvious from both Figs 3C and S6. The best performing s10 peptide in the library, H199Y/T203F, with a 54-fold increase in fluorescence compared to WT, is closely followed by H199Y/T203Y combined with P211D or N212 to A, K, D, R and I, respectively. (S7A Fig). The many advantageous substitutions to charged residues in the C-terminal flanking region outside the hotspot could potentially suggest a need for increased peptide charge. In the core region, additional beneficial substitutions include L201I and V206T which are confirmed in the absence of flanking regions in the s10_short_ format (S7B Fig).

### How well does array signal reflect binding affinity in solution?

The effect of the most beneficial substitutions was hypothesized to reflect an affinity increase at sub-saturation concentrations of LOO10-GFP (S8 Fig). On the other hand, intrinsic brightness of FP complexes at the wavelength of measurement would also affect apparent intensity. To address this issue, we chose to analyze the binding affinity and brightness, in solution, of the reconstituted GFP with the s10 WT peptide and three gain-of-function variants (H199Y, T203Y and H199Y/T203Y) in the short format and one loss-of-function variant in the long format (L207R). We examined whether their microarray signals correlated with brightness and/or affinity of the peptide variants upon reassembly with LOO10-GFP in solution.

**Fig 4:**
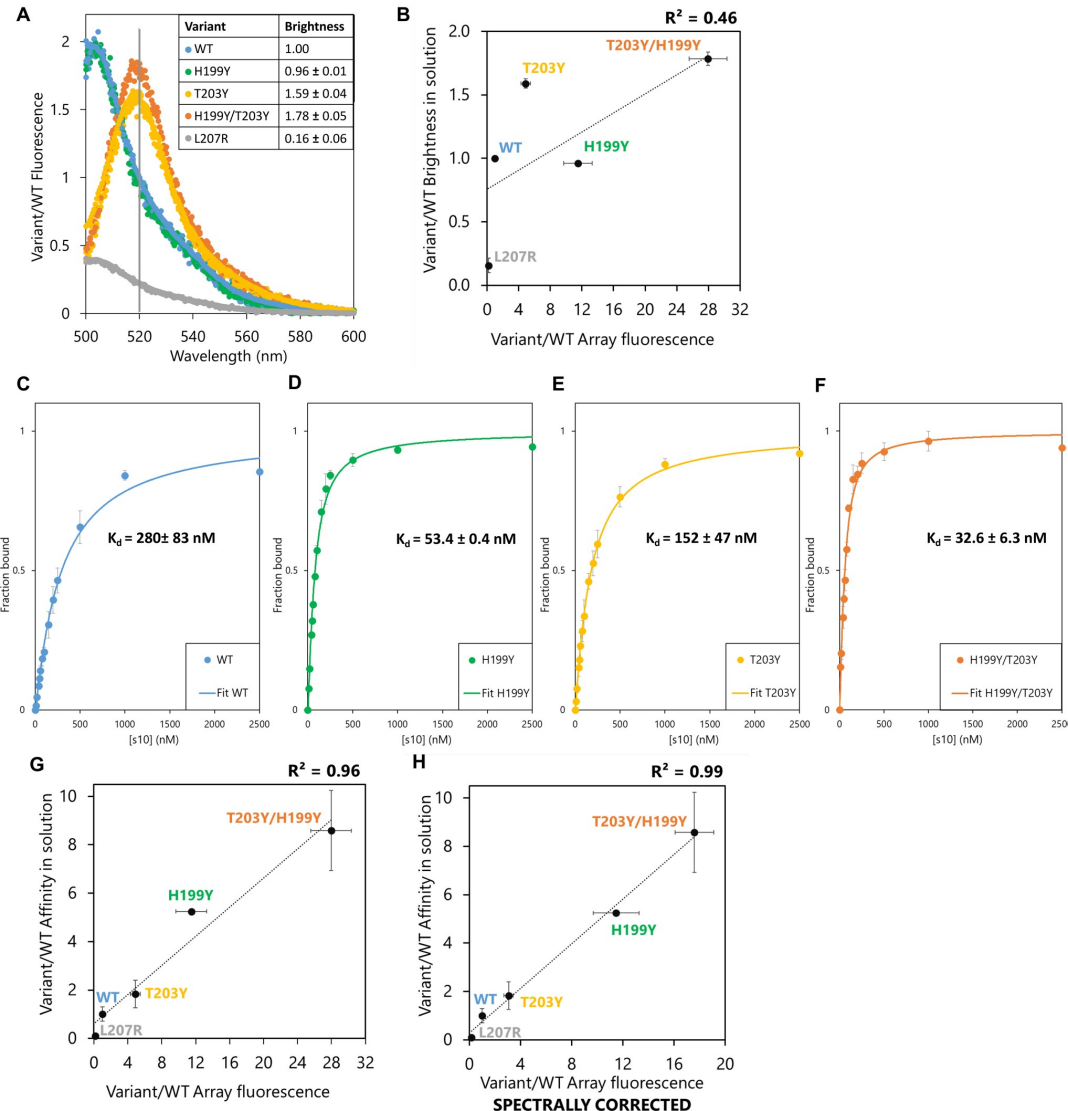
Brightness and affinity of selected s10 substitutions in solution. (A) Fluorescence emission spectra of LOO10-GFP saturated with each of the 5 s10 variant peptides and excited at 488 nm. The normalized to emission of WT complex at 520 nm (marked on the plot with a vertical line and indicated as brightness in the table insert). Spectra of 3 independent replicas for each peptide variant are overlaid in the plot. (B) Correlation between relative microarray fluorescence and relative brightness of the FP complexes in solution. (C-F) Binding isotherms of (C) WT, (D) H199Y (390ex/506em) and (E) T203Y, (F) H199Y/T203Y (495ex/520em) variants. Error bars represent standard deviation of 3 independent measurements. (G) Correlation between relative microarray fluorescence and relative affinity of the tested split fragments in solution agrees well with a linear model. (H) Combining the spectral and affinity properties in solution still explains the microarray fluorescence.

Upon complementation of LOO10-GFP at saturating concentrations of each s10 variant, we plotted individual relative emission spectra (Fig 4A) relative to WT. We observed no fluorescent complementation when LOO10-GFP was incubated with the L207R variant; the spectrum of this complex was unchanged relative to LOO10-GFP. S10 WT complemented LOO10-GFP with 5-fold increase in 520 nm signal, but no additional effect on brightness when introducing H199Y as a single substitution. T203Y variant caused a spectral shift of the emission maximum of the recovered FP complex from 506 to 520 nm, offering ~ 1.6-fold higher brightness of the T203Y variant compared to WT at 520 nm. H199Y in combination with T203Y increased the 520 emission by 1.8-fold compared to WT. The loss-of-function effect of L207R and the gain-of-function effects of T203Y variants were both captured by the microarray fluorescence of these variants. Still, brightness effects in solution could not explain the microarray data; a R^2^ < 0.5 being obtained when correlating the two datasets (Fig 4B). In particular, H199Y, with a 10-fold increase in microarray fluorescence as a single substitution, showed no brightness effects when saturating LOO10-GFP with this variant in solution.

Next, we titrated LOO10-GFP with increasing concentrations of s10 variant in solution and fitted the binding curves (Fig 4C–4F) to calculate dissociation constants, *K*_d_, as a measure of affinity between the fragments. The H199Y substitution showed a ~ 5-fold increase in affinity compared to WT, while the double substituted H199Y/T203Y had a ~ 9-fold increase. The ~ 2-fold increase in affinity of T203Y relative to WT previously reported for a 19-mer s10 [12] is replicated by our data. Competition experiments suggested that L207R variant binds LOO10-GFP very weakly and is almost fully displaced by a 4-fold lower concentration of WT variant (S9 Fig). We therefore approximated the affinity of L207R to be at least 10-fold weaker than WT.

Assuming a sub-saturation regime where *K*_d_ is greater than the LOO10-GFP concentration, the association constants, *K*_d_^-1^, should scale linearly with array fluorescence, see Methods. Under this assumption, affinity effects are more likely to explain the array fluorescence, although they do not take into account the spectral contributions (Fig 4G). Furthermore, taking the spectral shift for T203Y and H199Y/T203Y into account, the spectrally corrected microarray fluorescence offered a slightly better fit, although probably not significant (Fig 4H). Based on these experiments, we conclude that with affinities in the nM range, the signal intensity on peptide microarrays in this format faithfully reflects the binding affinity between the split fragments.

## Discussion

We have developed a precise, robust and accurate method for exploring the substitutional landscape for leave-one-out split fluorescent proteins, that has generated a comprehensive sequence-function map of a splitFP tag. Chemical synthesis of peptides in the library avoided time-consuming and bias-inducing steps like cloning, expression, purification or sequencing, that are usually required in genetic screens. By having full control over the s10 sequence on the microarray, we circumvented the limitations of the DNA codon table. Because double and triple substitutions in a given codon are rare, random mutagenesis will typically only generate a subset of mutations [21] and will never exhaustively sample double or triple amino acid substitutions. In the microarray setup it is possible to investigate every peptide in the library independently of Hamming distance from the starting sequence at the DNA level. Thus, libraries can be set up highly diverse (potentially including non-natural amino acid residues) offering a one-to-one picture of the entire functional/binding landscape, including low and medium performing sequences. In addition, because the LOO-FP chromophore is matured prior to our assay, we accessed high affinity s10 variants that might not be discoverable by multiplexed expression of the full-length proteins, where the chromophore cyclisation and oxidation is a prerequisite for fluorescence. Indeed, the H199Y substitution either does not show up or is identified as likely destabilizing in other GFP genetic screens [21,22]. It is an interesting possibility that this variant, while stabilizing the mature GFP, is disfavored in genetic screens because it does not stabilize the non-fluorescent immature precursor. Lastly, our assay proposed a direct measure of peptide binding in high-throughput, avoiding false positives caused by oligomeric fluorescent species that can appear in genetic selection experiments performed in cells [9].

We should point out that the interpretation of results in the microarray platform assumes a similar chemical yield of peptide across variants. The level of reproducibility between 12 replicas suggested intra-sequence synthesis yields are very similar. Inter-sequence yields were more difficult to assess, but the consistently lower signals when incorporating histidine in any part of s10 might suggest a reduced coupling yield when histidine is incorporated in the sequence. However, low histidine coupling yield was not observed in another study using microarrays from the same manufacturer [14].

In this analysis, we identified several interesting GFP strand 10 peptide substitutions and truncations. In particular, we note a double substitution, H199Y/T203F, which presented 54-fold higher microarray fluorescence compared to the WT sequence. We also found that the H199Y/T203Y s10 variant had almost 10-fold higher solution affinity compared to WT. Truncations of s10 to 11-mer or even 9-mer proved active on the peptide microarray and the 11-mer also effectively reconstituted fluorescence as a free peptide in solution. These short versions of s10 with the H199Y substitution could be readily used for *in vitro* applications, and could possibly be further investigated as non-interfering protein tags due to their small size.

For developing this screening platform, we used the split 10 FP system as a model, mainly due to its high affinity *in vitro*. Still, the LOO10-GFP efficiency *in cellulo* proved poor in previous studies [6,23]. For engineering improved *in cellulo* and *in vivo* tags using the microarray platform reported here, one could turn to the strand 7 and strand 11 LOO systems [23]. By incubating a microarray library with an immature LOO-FP, one could study what the sequence requirements would be for both binding and chromophore maturation. Indeed, β-strand-assisted chromophore maturation of LOO11-GFP on solid support has already been demonstrated [24].

Reaching saturation for every peptide could theoretically offer the possibility of detecting variants with intrinsic FP brightness improvement, desirable for many applications. A limitation in our study was that LOO10-GFP is a rather unstable protein with fairly low solubility, 2 μM being the maximum reliable concentration we could obtain for our microarray experiments. A more stable LOO10-GFP might be desirable, since titrating the microarray with increasing concentrations of LOO10-GFP could saturate all peptides and possibly allow plotting full binding curves for each peptide. This possibility was demonstrated in previous microarray screens [25,26]. In the absence of strand 10, on the other hand, a hydrophobic patch is exposed, thus mutations that stabilize LOO10-GFP, *e*.*g*. by making this surface more hydrophilic, would also likely decrease affinity to strand 10.

We believe the platform reported here can be generalized to many split fluorescent and luminescent proteins. Screening on microarrays may be limited by unspecific binding which is avoided for split systems that require specific complementation in order to function. Different color FPs could be studied, since microarray scanners employ excitation laser sources emitting in the blue, green and red spectrum regions and provide multiple emission filter options. Tuning protein concentrations to the sub-saturation regime is typically not difficult, however, requires a bright chromophore and a sensitive microarray reader. For reasons of sensitivity, assaying split proteins using this technology is most likely limited to fluorescence detection. Thus, studying split enzymes systems would likely require appropriate (insoluble) fluorescent product formation. One possibility is using internally quenched substrates, similar to those used in some protease assays [27].

Overall, full control over the desired substitutions on peptide microarrays makes them versatile alternatives to cell-based approaches for rational design of binders, massively parallel testing of computational design and benchmarking biophysical prediction methods.

## Methods

### Preparation of LOO10-GFP

We used an adapted method from [12]. Engineered plasmids of full-length circularly permuted superfolder GFP in pET-15b vectors were kindly provided by Steven Boxer, Stanford University. The full-length GFP was expressed in BL21 (DE3) cells in commonly used AB-LB growth media, where ampicillin (VWR) was added to a concentration of 100 μg/ml and glucose to a concentration of 1% (w/v). The starter culture was grown overnight at 37°C in LB medium, then expanded in AB-LB medium in a ratio of 1:100 starter culture to growth medium. This culture was grown at 37°C until an OD_600_ of ~0.6, induced with 1 mM isopropyl β-d-1-thiogalactopyranoside (Sigma) and agitated at 17°C overnight for protein expression. The cells were harvested on a Lynx 4000 (SORVALL) centrifuge at 20,000g for 30 minutes. The cell pellet was resuspended in lysis buffer 50 mM Hepes, 300 mM NaCl, pH 8 at 25 ml buffer per liter culture and sonicated in an UP2009 (Hielscher) for 8 cycles 30 sec pulse/30 sec pause. After one round of 13,000g centrifugation for 15 minutes, the supernatant was poured onto a Ni-NTA column (GE Healthcare) equilibrated with lysis buffer. Three column volumes of lysis buffer supplemented with 20 mM imidazole (Merck) was used for washing, and the same buffer supplemented 200 mM imidazole was used to elute the protein. Imidazole was removed by dialyzing the sample through a Spectra/Por 3.5 kDa membrane into lysis buffer overnight at 4°C.

The loop between strand 10 and 11 was digested using a 0.5 μM trypsin (DIFCO) solution. The reaction contained full length circularly permuted GFP and 1% (M/M) trypsin in cleavage buffer 50 mM Tris, 20 mM CaCl_2_ (Merck), pH 8. The tube was incubated for 30 minutes at room temperature, before the trypsin was inhibited with protease inhibitor phenylmethylsulfonyl fluoride (Sigma), at a final concentration of 1 mM. The mix was then precipitated in a 60% ammonium sulfate solution and centrifuged at 5000g for 10 minutes at 4°C. The pellet was dissolved in denaturation buffer (50 mM Hepes, 50 mM NaCl, 6 M GdnHCl, pH 8) and loaded on a Superdex 75 10/300 GL (GE Healthcare) size exclusion column to remove s10. LOO10-GFP was eluted isocratically in denaturation buffer at 0.6 ml/min. Fractions with absorption A_447_ > 0.1 (1 cm path length) were pooled together and stored at 4°C until usage. For quantitative measurements and microarray experiments, LOO10-GFP was refolded by desalting on an Illustra NAP5 column (GE Healthcare) equilibrated with assay buffer 50 mM Hepes, 100 mM NaCl, 0.1% v/v Tween20, pH 8 using the manufacturer’s protocol.

### Synthetic peptides

Chemically synthesized s10 peptides NHYLSTQTVLS (WT), NYYLSTQTVLS (H199Y), NHYLSYQTVLS (T203Y), NYYLSYQTVLS (H199Y/T203Y), LPDNHYLSTQTVRSKDPNE (L207R) were purchased from Schafer-N or TAG Copenhagen with >95% purity.

### Fluorescence spectroscopy

Spectral measurements were performed by mixing 30 nM LOO10-GFP with 3 μM (100 fold excess) of each s10 peptide variant (WT, L207R, H199Y, T203Y or H199Y/T203Y) and incubated ~ 1 hour in assay buffer for full complementation. Fluorescence emission spectra were recorded by exciting samples at 488 nm (5 nm slit) and a 5 nm emission slit in the 495–600 nm range on a PerkinElmer LS 55 Luminescence Spectrometer at 25°C. Spectra were taken in 3 independent replicas for each peptide variant.

Affinity measurements were performed by mixing 50 nM LOO10-GFP with solutions of increasing concentrations of each s10 peptide variant WT, H199Y, T203Y or H199Y/T203Y (0, 10, 20, 40, 50, 60, 80, 100, 150, 200, 250, 500, 1000, 2500 nM) and incubating overnight at 4°C for equilibration. Fluorescence of each sample was measured at 390/506 nm for T203 variants and at 495/520 nm for Y203 variants, at 25°C, excitation slit 12 nm, emission slit 20 nm and 10s integration time. The affinity experiments were performed in 3 independent replicas for each peptide variant. Fluorescence data for each curve were fit to a quadratic equation:

$$F=F_{0}+{(F}_{max}-F_{0})*\frac{\left((G+P+K_{d})-\sqrt{{\left(G+P+K_{d}\right)}^{2}-4*G*P}\right)}{2*G}$$

where *F*_0_ was background fluorescence (FU), *F*_max_ was fluorescence at saturation (FU), *G* was LOO10-GFP concentration (nM), *P* is s10 peptide concentration (nM) and *K*_d_ is the binding affinity between the two fragments (nM). All fits were performed in OriginPro 2017.

### Absorption spectroscopy

Concentration of LOO10-GFP was estimated by measuring absorbance at 447 nm and using its extinction coefficient at 0.1 M NaOH of *Ɛ*_447_ = 44,100 M^-1^ cm^-1^ [12]. The s10 peptides were dissolved in assay buffer and the predicted *Ɛ*_280_ based on tryptophan and tyrosine content was used for concentration determination [28]. All absorbance measurements were done on a Perkin Elmer Lambda 35 Spectrophotometer.

### Microarray incubation and data analysis

The peptide library was synthesized on a single chip with 12 identical sectors, each containing 15,301 peptide spots of 20 x 20 μm, 183,612 peptide spots in total. Out of these, 12,544 peptide variants x 12 = 150,528 spots were analyzed for this report. The remaining 2757 spots were part of another project and are not reported here.

Before incubation with LOO10-GFP, the array surface was hydrated with assay buffer for 10 minutes at 25°C under shaking. After removal of the buffer, 4 mL solution of 2 μM LOO10-GFP in assay buffer was added to the array and incubated overnight (~ 18 hours) at 4°C under shaking. The array was washed two times in assay buffer at room temperature and dried under a gentle stream of N_2_. The array was imaged on an InnoScan 1100 AL (INNOPSYS) microarray laser scanner at 488 nm excitation and with a 520±5 nm emission bandpass filter. Images were collected at low laser power (5 mW) with gain of 20, at 1 μm resolution. Fluorescence data were extracted from images using ImageJ MicroArray Profile plugin Version 3.1. with rectangle ROIs of 20 x 20 μm and assigned to each peptide variant spot.

Any outliers caused by dust or other contaminations were estimated by observing the distribution of the 12 replicas for each peptide. We chose a conservative cutoff for outlier removal, since contaminants should be very bright compared to a regular high signal. Thus, all the values less than 6 Median Absolute Deviation (MAD) above the median for each peptide were taken into further analysis, while 299 (<2%) were eliminated as outliers. MAD is generally not influenced by outliers, so the observation that the standard deviation after outlier removal is similar to MAD before outlier removal (S10 Fig**)** demonstrates that most outliers were successfully eliminated.

As each of the 12 sectors were, in principle, identical, the average sector signal should be the same. However, the fact that average brightness shifted in a consistent gradient across the slide suggested an artificial global variation (S11 Fig). This could be due to inhomogeneity of the functional surface or inhomogeneity of buffer component allocation during incubation or washing steps. However, when plotting all fluorescent signals of each sector against the others, we observed that the variant libraries in all 12 sector replica correlated well with each other, with Pearson coefficients > 0.94 (S3 Fig), therefore we considered appropriate normalizing the mean fluorescence of each sector to the mean fluorescence of the microarray. When examining the impact of normalization on data quality, we observed that the mean variant fluorescence of 12 replicas was maintained constant after normalization (S12A Fig), while the standard deviation values dropped ~ 2 fold (S12B Fig). Thus, the normalization procedure did not quantitatively change the fluorescence signals of variants, but only made the data more precise. The array background was removed for each peptide by subtracting the mean fluorescence of blank spots in each sector. All negative/zero fluorescence values after background removal were replaced with the value +1, to avoid issues at log2 transformation. We measured the mean fluorescence and standard deviation for the s10_long_ WT peptide at 741 ± 270 fluorescence units (FU) for non-normalized data and 741 ± 64 FU for normalized data.

Substitutional tolerance at each s10 position was calculated by counting the number of substitutions in that position which cause a change in fluorescence, either gain-of-function or loss-of-function (as defined previously). We subtracted this number from the total 19 substitutions at each position.

If not stated otherwise, fluorescence values were outlier-and-background-removed normalized mean variant fluorescence of 12 replica. From these values (henceforth denoted simply as fluorescence) all plots are generated, except heatmaps and variant effect plots where we used log2(fluorescence), and the experimental validation dataset where we used relative variant fluorescence against WT *i*.*e*. variant fluorescence/WT fluorescence.

### Thermodynamic description of microarray binding and spectral correction

The equilibrium between LOO10-GFP, *P*, immobile s10 peptide, *S*, and the fluorescent complex, *PS*, gives the fraction of fluorescent chromophores per microarray spot:

$$F\propto \frac{\left[PS\right]}{{\left[S\right]}_{0}}=\frac{1}{1+K_{d}/\left[P\right]}\approx \frac{\left[P\right]}{K_{d}}$$

We assume this to be proportional to the observed fluorescence, *F*, and that all spots have the same amount of peptide [*S*]_0_ = [*S*]+[*PS*]. The last approximation describes the sub-saturation regime *K_d_*>[*P*].

For spectral correction, the microarray fluorescence of T203Y and H199Y/T203Y is simply divided by the spectral shift factor of 1.6 determined from Fig 4A.

## Supporting information

S1 Fig Preparation and quality control of LOO10-GFP. (A) Five μg cp-sfGFP digested with different trypsin amounts: 1, 5, 10, 20% molar ratio of trypsin to GFP. Rightmost lane is cp-sfGFP not digested by trypsin. Digestion for 30 minutes, stopped by addition of PMSF to a final concentration of 1 mM. (B) Five μg cp-sfGFP digested with 1% molar ratio of trypsin to GFP for different time periods: 0, 5, 10, 20, 30 and 45 minutes. 0 minutes is before addition of trypsin. The digestion was stopped by addition of PMSF to a final concentration of 1 mM. (C) Reassembly of synthetic strands (s10 Long, Medium, Short and Negative) with LOO10-GFP. 100nM LOO10-GFP was mixed with 25X excess of the respective peptide in HEPES buffer at 25°C. The reassembly was tracked by measuring the chromophore fluorescence as a function of time, at 390 nm excitation and 506 nm emission.
(DOCX)

S2 Fig Preliminary microarray with 3498 peptide features of 40 x 40 μm was incubated with 2 μM LOO10-GFP overnight at 4°C. Imaging of the microarray was performed at 488 nm excitation and 520 nm emission with 1 μm resolution on an InnoScan 1100 fluorescence scanner. The 8Bit image was analyzed and background fluorescence (unused fields) was substracted for each peptide. (A) Effect of s10 peptide length and C-terminal linkers. Intensity values are background-subtracted absolute fluorescence values and standard deviations are calculated over n = 50 replica for each variant. (B) Fluorescence heatmap of single substitutions of s10 long with A, D, G, R, T, V and Y residues. Color key: Yellow–WT- like fluorescence; Red–loss of function substitutions; Green–gain of function substitutions. Fluorescence represents background-subtracted absolute fluorescence values; relative standard deviations over n = 10 replica for each variant are < 10%.
(DOCX)

S3 Fig Correlations between the 12 sectors. Correlation of variant fluorescence between all 12 sector replicas, after outlier removal but before sector normalization. The Pearson correlation coefficients are indicated in the upper matrix.
(DOCX)

S4 Fig Linker effects in the short format. Correlations between signals from substitutions in s10_short_ with different linkers: no linker, positive GKGSKSG (gk7) linker, negative GEGSESG (ge7) linker, and neutral GSGSGSG (gs7) linker. The Pearson correlation coefficients between the different substitutional libraries are indicated in the upper matrix. Note the increased scatter for peptides without linker compared to peptides with (chart bottom row—and Pearson coefficients top row).
(DOCX)

S5 Fig Left- and right-shifting effects. s10_short_ versus s10_shortL_ and s10_shortR_. All substitution variants of the short format with ge7 (red), gk7 (green), gs7 (dark blue) and no linker (light blue) on the x-axes compared to the same substitution and linker variants of the (A) shortL and (B) shortR formats on the y-axis. Variants of the terminus positions that are not present in both formats are marked with magenta or grey crosses.
(DOCX)

S6 Fig Effect of context on the fluorescence of substitutional variants. Comparing single (WT context), double (H199Y, T203Y, T203I, L207V and L207R contexts) and triple (H199Y/T203Y context) substitutions from s10_long_ WT. In each context, the jitter points are colored based on the position substituted, resulting in 20 points of the same color per position. Each variant is presented as mean over 12 replica ± one standard deviation as error bar. The dashed lines delimit the “WT interval” defined as mean WT fluorescence ± 4.3 standard deviations (corresponding to 2 times the un-normalized standard deviations). The interval above WT is considered GoF (gain-of-function), while the interval below LoF (loss-of-function).
(DOCX)

S7 Fig Top fluorescing variants. (A) Box plot of top 9 highest fluorescing variants of the long format, representing the core sequence and flanking regions. (B) Box plot of top 5 highest fluorescing variants the short-gk7 format, representing only the core sequence. In both (A) and (B), each box indicates the distribution of absolute fluorescence of each variant across the 12 replica. Box plots of some single substitutions and the H199Y/T203Y double substitution are added for comparison.
(DOCX)

S8 Fig Proposed model for interaction at the peptide microarray surface. Higher microarray fluorescence at peptide field A/C than at peptide field B/D can occur due to: (left) Sub-saturation conditions: Higher affinity of peptide A than peptide B to LOO10-GFP, resulting in more complemented FP molecules on peptide field A than B or (right) Saturation conditions: Higher brightness of FP complex C than D, at equal number of complemented FP molecules on peptide field C and D.
(DOCX)

S9 Fig Competition studies with L207R variant. Time resolved fluorescence of association between 100 nM LOO10-GFP and 2.5 μM s10_short_ WT, with (blue curve) or without (red curve) pre-incubation with 10 μM s10_long_ L207R.
(DOCX)

S10 Fig Quality control of the microarray data after outlier removal. Scatter plot comparing the median absolute deviation (MAD) across 12-replica for each variant before outlier removal and the standard deviation (SD) across 12-replica for the same variant after outlier removal.
(DOCX)

S11 Fig Fluorescence across the 12 microarray sectors. Heatmap of mean fluorescence per sector, before normalization. Color key: Linear gradient between grey for dim sectors and green for bright sectors.
(DOCX)

S12 Fig Quality control of the microarray data after cleaning. (A) Mean and (B) SD across the 12 replica for each variant before normalization plotted against the same quantity after normalization.
(DOCX)

S1 Table Overview of the composition of the peptide library. Strand 10 substitutional variants and controls synthesized on the microarray. The linker sequences are gs2: GS; gs7: GSGSGSG, gk7: GKGSKSG and ge7: GEGSESG.
(DOCX)
