# Supplementary material for: Substitutional landscape of a split fluorescent protein fragment using high-density peptide microarrays
Source: PLoS One. 2021 Feb 3;16(2):e0241461. doi: 10.1371/journal.pone.0241461 (PMC7857580; doi:10.1371/journal.pone.0241461)
Supplement: S1 Fig — (A) Five μg cp-sfGFP digested with different trypsin amounts: 1, 5, 10, 20% molar ratio of trypsin to GFP. Rightmost lane is cp-sfGFP not digested by trypsin. Digestion for 30 minutes, stopped by addition of PMSF to a final concentration of 1 mM. (B) Five μg cp-sfGFP digested with 1% molar ratio of trypsin to GFP for different time periods: 0, 5, 10, 20, 30 and 45 minutes. 0 minutes is before addition of trypsin. The digestion was stopped by addition of PMSF to a final concentration of 1 mM. (C) Reassembly of synthetic strands (s10 Long, Medium, Short and Negative) with LOO10-GFP. 100nM LOO10-GFP was mixed with 25X excess of the respective peptide in HEPES buffer at 25°C. The reassembly was tracked by measuring the chromophore fluorescence as a function of time, at 390 nm excitation and 506 nm emission. (DOCX) [file pone.0241461.s001.docx]

***
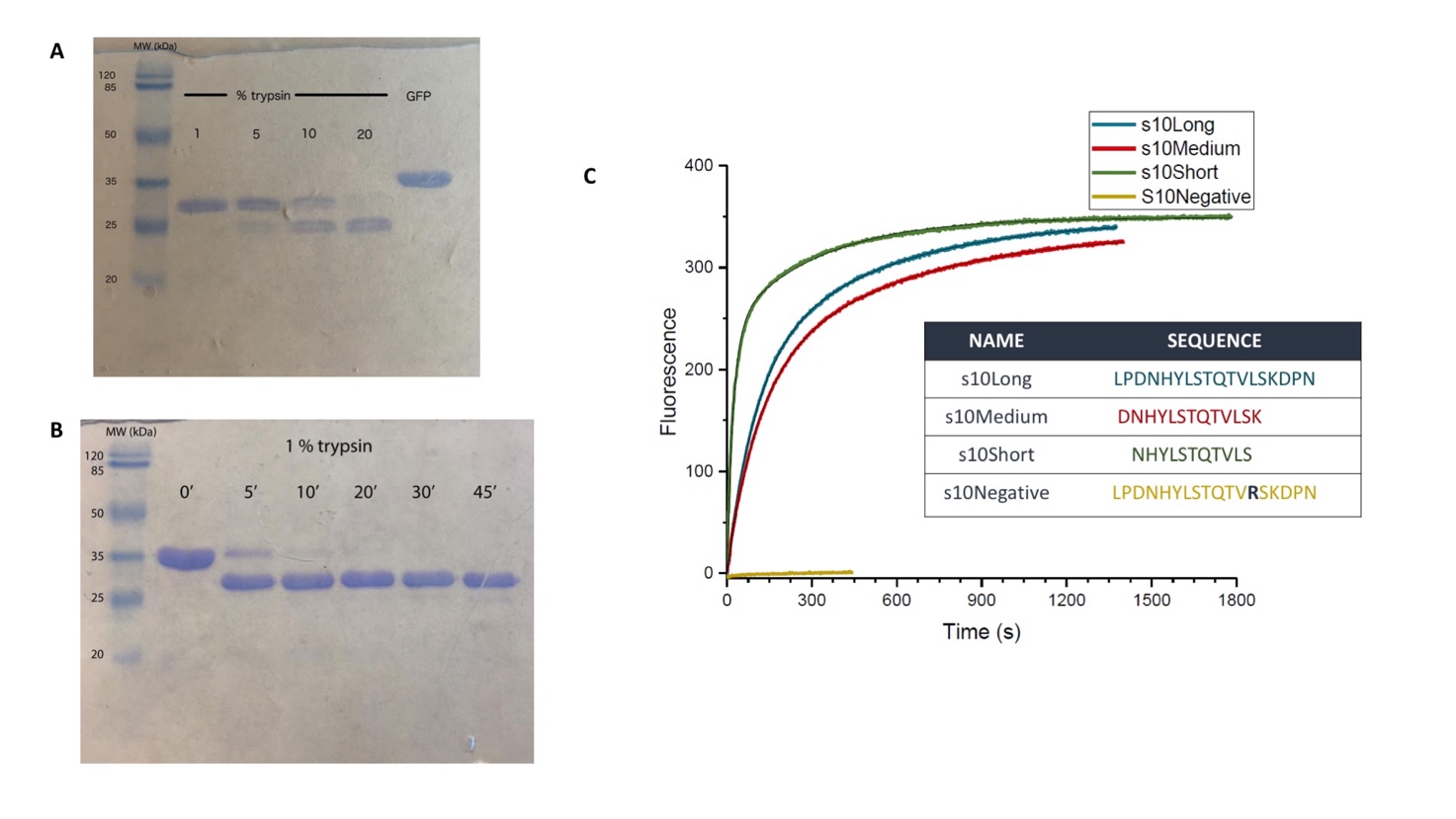
***

**S1 Fig. Preparation and quality control of LOO10-GFP** (A) Five μg cp-sfGFP digested with different trypsin amounts: 1, 5, 10, 20 % molar ratio of trypsin to GFP. Rightmost lane is cp-sfGFP not digested by trypsin. Digestion for 30 minutes, stopped by addition of PMSF to a final concentration of 1 mM. (B) Five μg cp-sfGFP digested with 1 % molar ratio of trypsin to GFP for different time periods: 0, 5, 10, 20, 30 and 45 minutes. 0 minutes is before addition of trypsin. The digestion was stopped by addition of PMSF to a final concentration of 1 mM. (C) Reassembly of synthetic strands (s10 Long, Medium, Short and Negative) with LOO10-GFP. 100nM LOO10-GFP was mixed with 25X excess of the respective peptide in HEPES buffer at 25°C. The reassembly was tracked by measuring the chromophore fluorescence as a function of time, at 390 nm excitation and 506 nm emission.
