# Supplementary material for: Substitutional landscape of a split fluorescent protein fragment using high-density peptide microarrays
Source: PLoS One. 2021 Feb 3;16(2):e0241461. doi: 10.1371/journal.pone.0241461 (PMC7857580; doi:10.1371/journal.pone.0241461)
Supplement: S2 Fig — Imaging of the microarray was performed at 488 nm excitation and 520 nm emission with 1 μm resolution on an InnoScan 1100 fluorescence scanner. The 8Bit image was analyzed and background fluorescence (unused fields) was substracted for each peptide. (A) Effect of s10 peptide length and C-terminal linkers. Intensity values are background-subtracted absolute fluorescence values and standard deviations are calculated over n = 50 replica for each variant. (B) Fluorescence heatmap of single substitutions of s10 long with A, D, G, R, T, V and Y residues. Color key: Yellow–WT- like fluorescence; Red–loss of function substitutions; Green–gain of function substitutions. Fluorescence represents background-subtracted absolute fluorescence values; relative standard deviations over n = 10 replica for each variant are < 10%. (DOCX) [file pone.0241461.s002.docx]

*
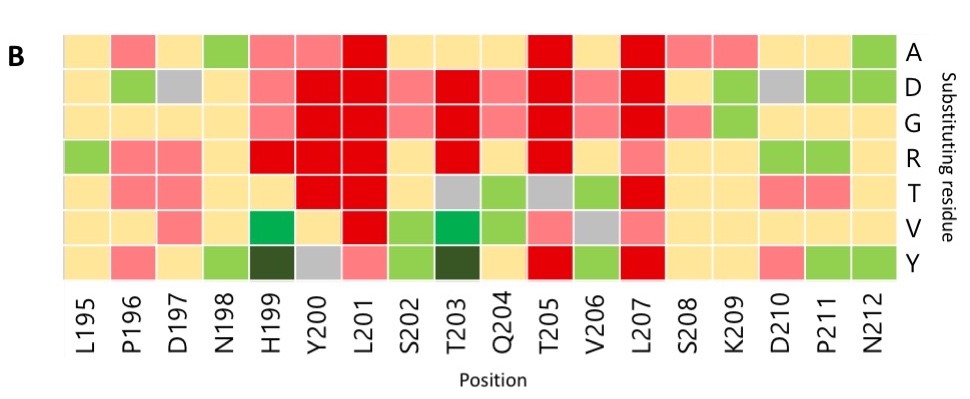
*

*
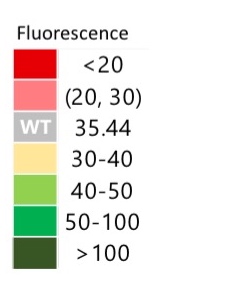
*

*
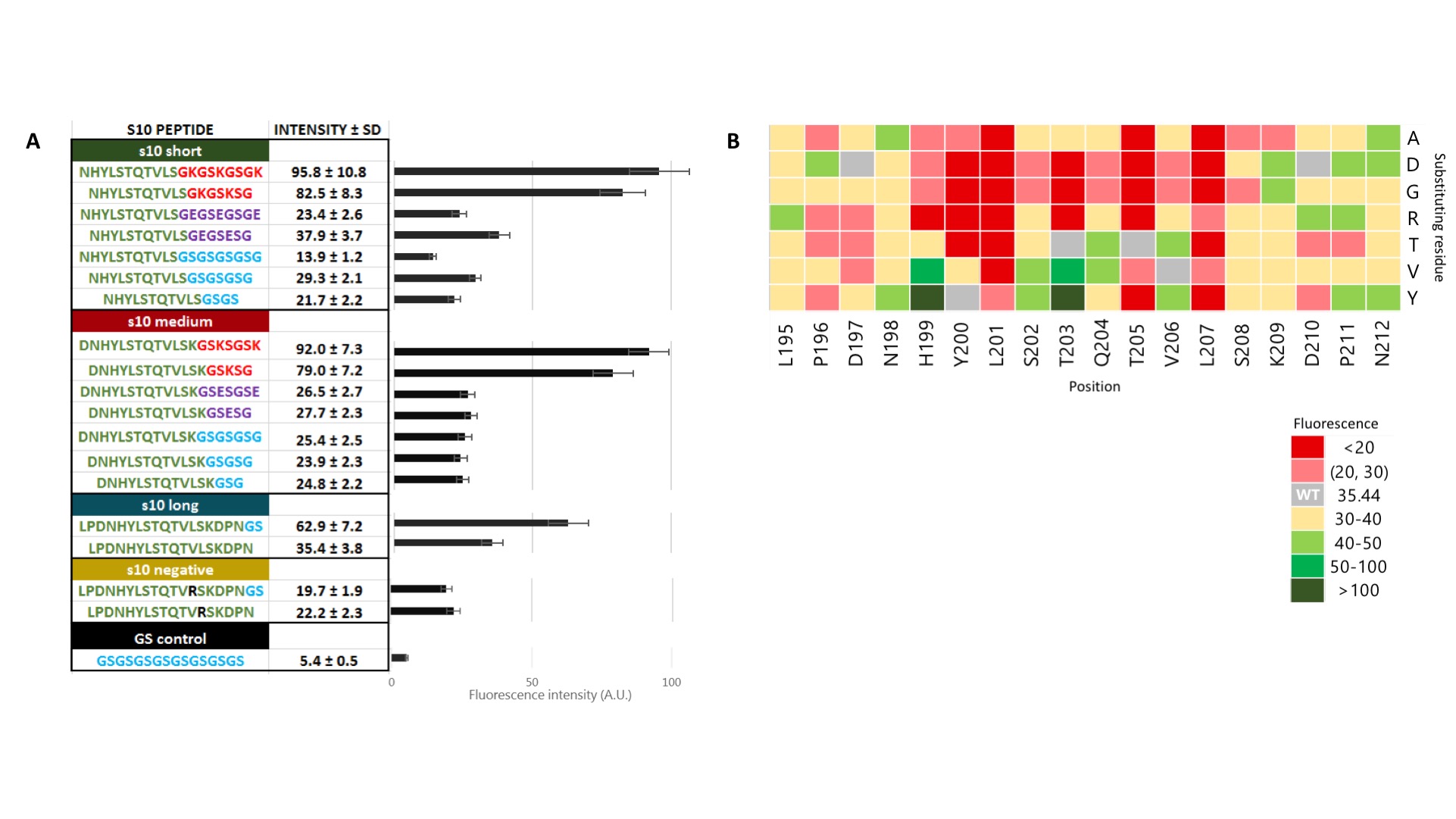
*

**S2 Fig. Preliminary microarray with 3498 peptide features of 40 x 40 µm was incubated with 2 µM LOO10-GFP overnight at 4°C.** Imaging of the microarray was performed at 488 nm excitation and 520 nm emission with 1 µm resolution on an InnoScan 1100 fluorescence scanner. The 8Bit image was analyzed and background fluorescence (unused fields) was substracted for each peptide. (A) Effect of s10 peptide length and C-terminal linkers. Intensity values are background-subtracted absolute fluorescence values and standard deviations are calculated over n = 50 replica for each variant. (B) Fluorescence heatmap of single substitutions of s10 long with A, D, G, R, T, V and Y residues. Color key: Yellow – WT- like fluorescence; Red – loss of function substitutions; Green – gain of function substitutions. Fluorescence represents background-subtracted absolute fluorescence values; relative standard deviations over n = 10 replica for each variant are < 10%.
