# Supplementary material for: Substitutional landscape of a split fluorescent protein fragment using high-density peptide microarrays
Source: PLoS One. 2021 Feb 3;16(2):e0241461. doi: 10.1371/journal.pone.0241461 (PMC7857580; doi:10.1371/journal.pone.0241461)
Supplement: S3 Fig — Correlation of variant fluorescence between all 12 sector replicas, after outlier removal but before sector normalization. The Pearson correlation coefficients are indicated in the upper matrix. (DOCX) [file pone.0241461.s003.docx]

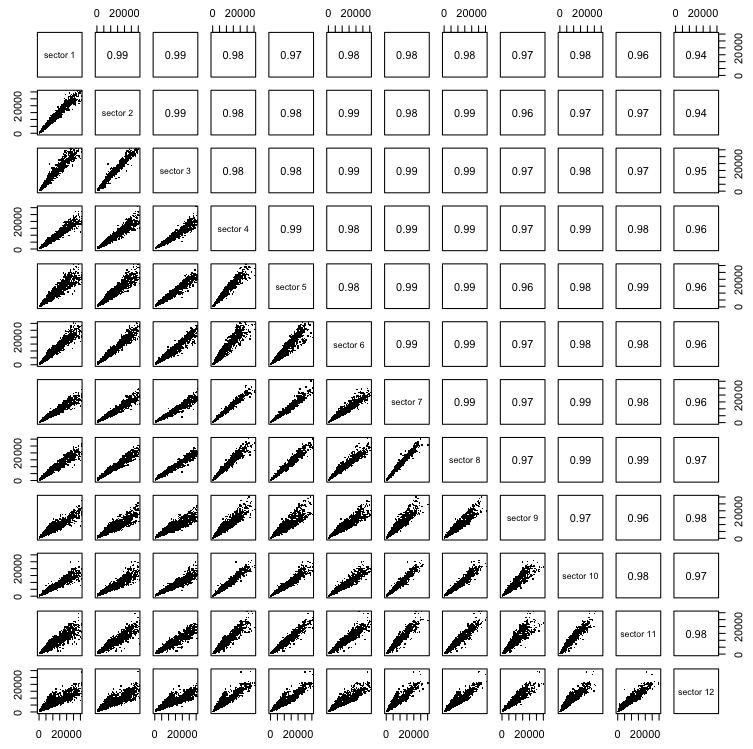


**S3 Fig. Correlations between the 12 sectors.** Correlation of variant fluorescence between all 12 sector replicas, after outlier removal but before sector normalization. The Pearson correlation coefficients are indicated in the upper matrix.
