# Supplementary material for: Substitutional landscape of a split fluorescent protein fragment using high-density peptide microarrays
Source: PLoS One. 2021 Feb 3;16(2):e0241461. doi: 10.1371/journal.pone.0241461 (PMC7857580; doi:10.1371/journal.pone.0241461)
Supplement: S4 Fig — Correlations between signals from substitutions in s10short with different linkers: no linker, positive GKGSKSG (gk7) linker, negative GEGSESG (ge7) linker, and neutral GSGSGSG (gs7) linker. The Pearson correlation coefficients between the different substitutional libraries are indicated in the upper matrix. Note the increased scatter for peptides without linker compared to peptides with (chart bottom row—and Pearson coefficients top row). (DOCX) [file pone.0241461.s004.docx]

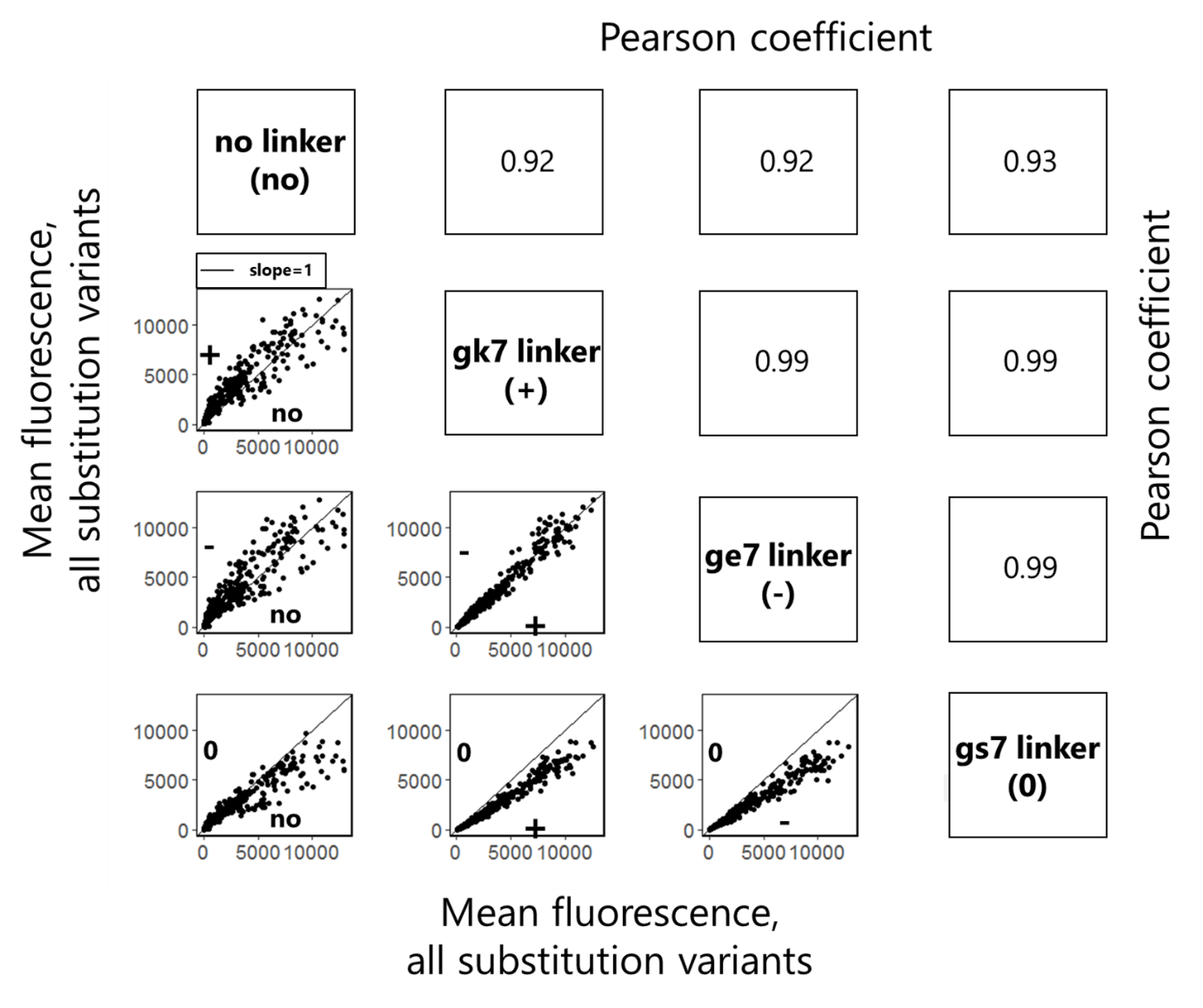


**S4 Fig. Linker effects in the short format.**  Correlations between signals from substitutions in s10_short_ with different linkers: no linker, positive GKGSKSG (gk7) linker, negative GEGSESG (ge7) linker, and neutral GSGSGSG (gs7) linker. The Pearson correlation coefficients between the different substitutional libraries are indicated in the upper matrix. Note the increased scatter for peptides without linker compared to peptides with (chart bottom row - and Pearson coefficients top row).
