# Supplementary material for: Substitutional landscape of a split fluorescent protein fragment using high-density peptide microarrays
Source: PLoS One. 2021 Feb 3;16(2):e0241461. doi: 10.1371/journal.pone.0241461 (PMC7857580; doi:10.1371/journal.pone.0241461)
Supplement: S5 Fig — s10short versus s10shortL and s10shortR. All substitution variants of the short format with ge7 (red), gk7 (green), gs7 (dark blue) and no linker (light blue) on the x-axes compared to the same substitution and linker variants of the (A) shortL and (B) shortR formats on the y-axis. Variants of the terminus positions that are not present in both formats are marked with magenta or grey crosses. (DOCX) [file pone.0241461.s005.docx]

***
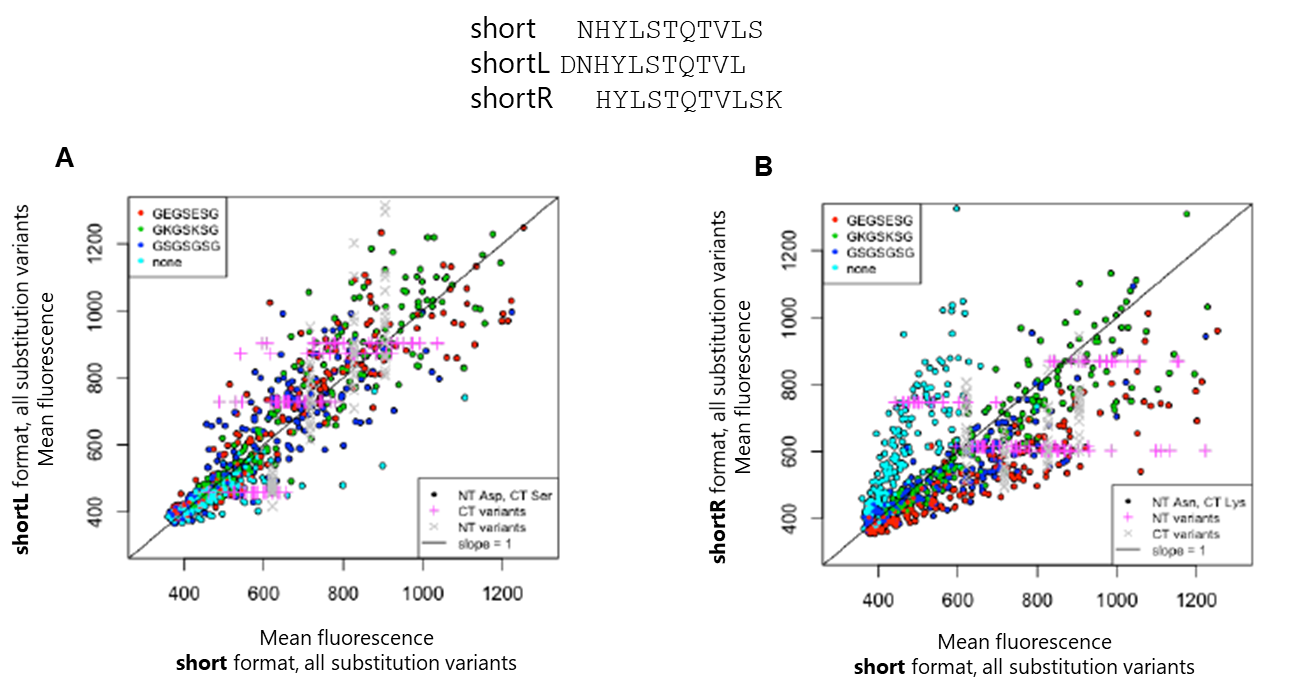
***

**S5 Fig. Left- and right-shifting effects.** s10_short_ versus s10_shortL_ and s10_shortR_**.** All substitution variants of the short format with ge7 (red), gk7 (green), gs7 (dark blue) and no linker (light blue) on the x-axes compared to the same substitution and linker variants of the (A) shortL and (B) shortR formats on the y-axis. Variants of the terminus positions that are not present in both formats are marked with magenta or grey crosses.
