# Supplementary material for: Substitutional landscape of a split fluorescent protein fragment using high-density peptide microarrays
Source: PLoS One. 2021 Feb 3;16(2):e0241461. doi: 10.1371/journal.pone.0241461 (PMC7857580; doi:10.1371/journal.pone.0241461)
Supplement: S6 Fig — Comparing single (WT context), double (H199Y, T203Y, T203I, L207V and L207R contexts) and triple (H199Y/T203Y context) substitutions from s10long WT. In each context, the jitter points are colored based on the position substituted, resulting in 20 points of the same color per position. Each variant is presented as mean over 12 replica ± one standard deviation as error bar. The dashed lines delimit the “WT interval” defined as mean WT fluorescence ± 4.3 standard deviations (corresponding to 2 times the un-normalized standard deviations). The interval above WT is considered GoF (gain-of-function), while the interval below LoF (loss-of-function). (DOCX) [file pone.0241461.s006.docx]

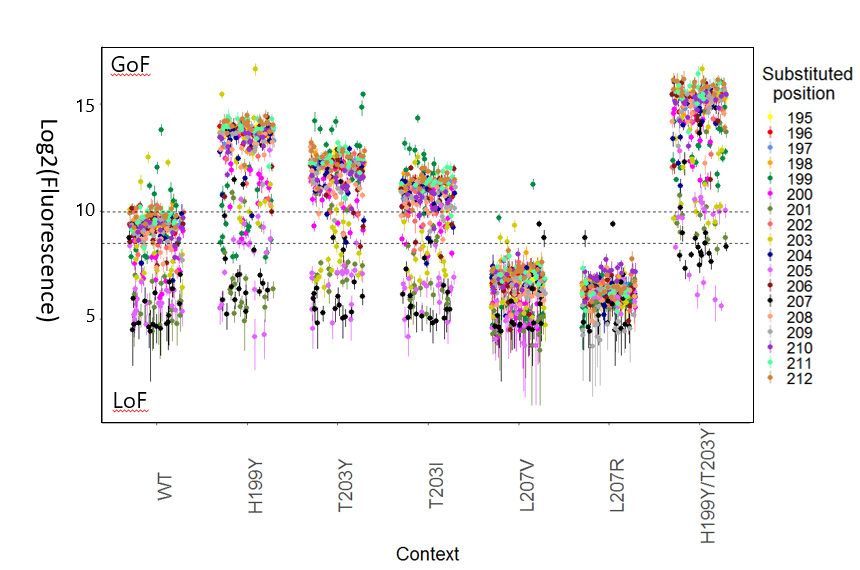


**S6 Fig. Effect of context on the fluorescence of substitutional variants**. Comparing single (WT context), double (H199Y, T203Y, T203I, L207V and L207R contexts) and triple (H199Y/T203Y context) substitutions from s10_long_ WT. In each context, the jitter points are colored based on the position substituted, resulting in 20 points of the same color per position. Each variant is presented as mean over 12 replica ± one standard deviation as error bar. The dashed lines delimit the “WT interval” defined as mean WT fluorescence ± 4.3 standard deviations (corresponding to 2 times the un-normalized standard deviations). The interval above WT is considered GoF (gain-of-function), while the interval below LoF (loss-of-function).
