# Supplementary material for: Substitutional landscape of a split fluorescent protein fragment using high-density peptide microarrays
Source: PLoS One. 2021 Feb 3;16(2):e0241461. doi: 10.1371/journal.pone.0241461 (PMC7857580; doi:10.1371/journal.pone.0241461)
Supplement: S7 Fig — (A) Box plot of top 9 highest fluorescing variants of the long format, representing the core sequence and flanking regions. (B) Box plot of top 5 highest fluorescing variants the short-gk7 format, representing only the core sequence. In both (A) and (B), each box indicates the distribution of absolute fluorescence of each variant across the 12 replica. Box plots of some single substitutions and the H199Y/T203Y double substitution are added for comparison. (DOCX) [file pone.0241461.s007.docx]

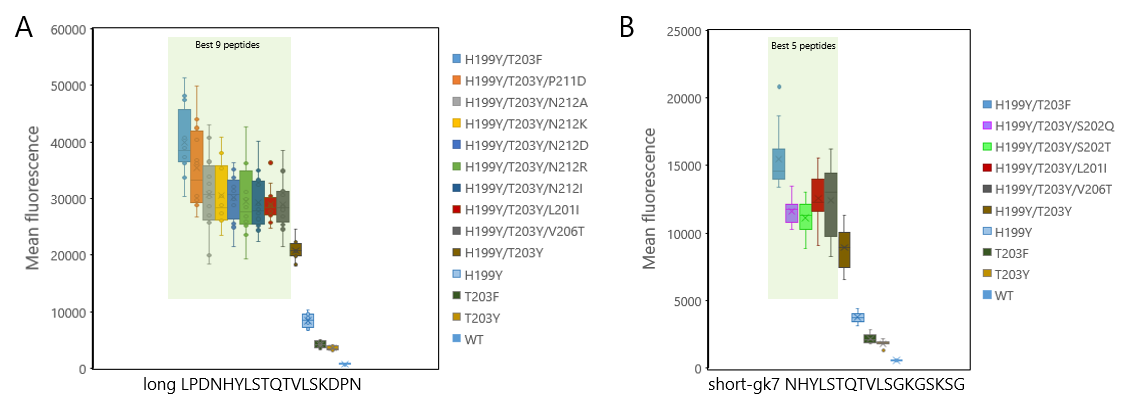


**S7 Fig. Top fluorescing variants.** (A) Box plot of top 9 highest fluorescing variants of the long format, representing the core sequence and flanking regions. (B) Box plot of top 5 highest fluorescing variants the short-gk7 format, representing only the core sequence. In both (A) and (B), each box indicates the distribution of absolute fluorescence of each variant across the 12 replica. Box plots of some single substitutions and the H199Y/T203Y double substitution are added for comparison.
