# Supplementary material for: Substitutional landscape of a split fluorescent protein fragment using high-density peptide microarrays
Source: PLoS One. 2021 Feb 3;16(2):e0241461. doi: 10.1371/journal.pone.0241461 (PMC7857580; doi:10.1371/journal.pone.0241461)
Supplement: S8 Fig — Higher microarray fluorescence at peptide field A/C than at peptide field B/D can occur due to: (left) Sub-saturation conditions: Higher affinity of peptide A than peptide B to LOO10-GFP, resulting in more complemented FP molecules on peptide field A than B or (right) Saturation conditions: Higher brightness of FP complex C than D, at equal number of complemented FP molecules on peptide field C and D. (DOCX) [file pone.0241461.s008.docx]

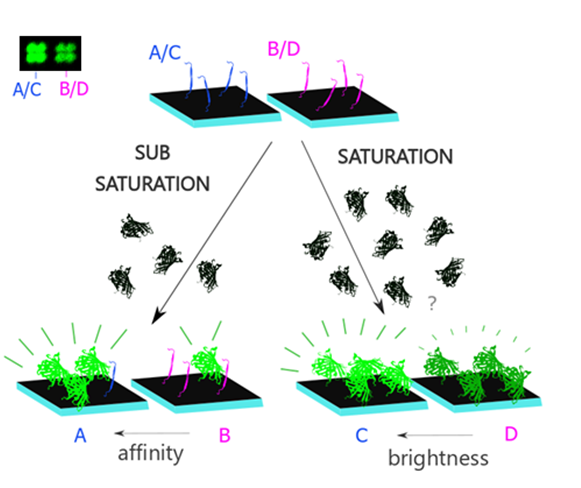


**S8 Fig. Proposed model for interaction at the peptide microarray surface.** Higher microarray fluorescence at peptide field A/C than at peptide field B/D can occur due to: (left) Sub-saturation conditions: Higher affinity of peptide A than peptide B to LOO10-GFP, resulting in more complemented FP molecules on peptide field A than B or (right) Saturation conditions: Higher brightness of FP complex C than D, at equal number of complemented FP molecules on peptide field C and D.
