# Supplementary material for: Substitutional landscape of a split fluorescent protein fragment using high-density peptide microarrays
Source: PLoS One. 2021 Feb 3;16(2):e0241461. doi: 10.1371/journal.pone.0241461 (PMC7857580; doi:10.1371/journal.pone.0241461)
Supplement: S9 Fig — Time resolved fluorescence of association between 100 nM LOO10-GFP and 2.5 μM s10short WT, with (blue curve) or without (red curve) pre-incubation with 10 μM s10long L207R. (DOCX) [file pone.0241461.s009.docx]

***
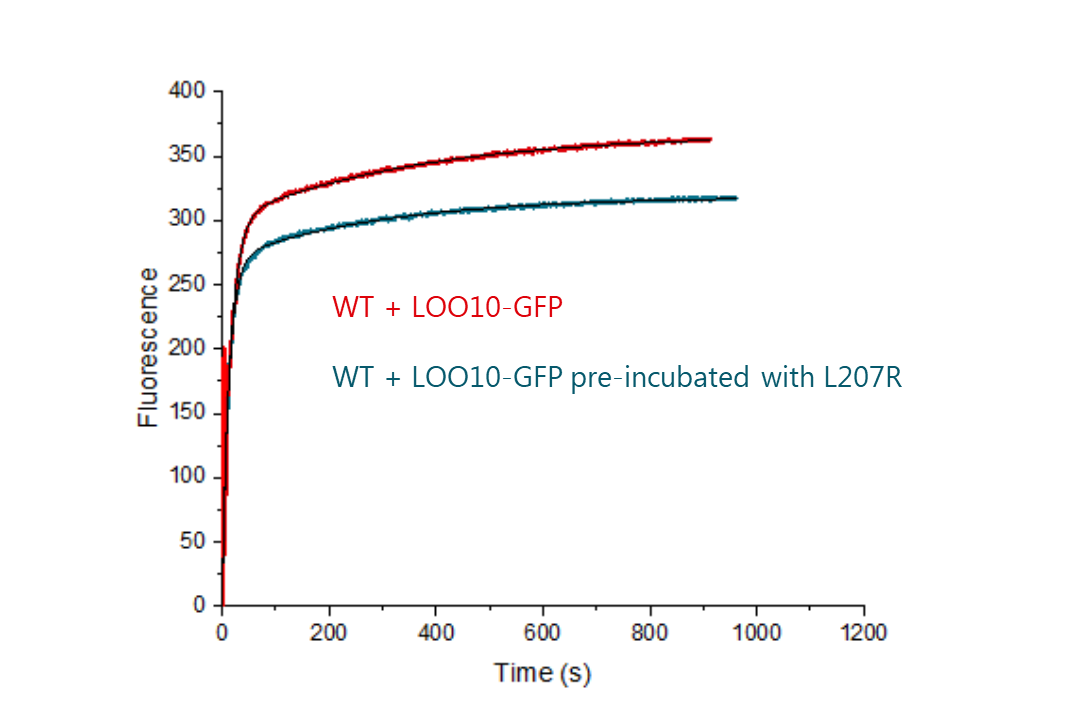
***

**S9 Fig. Competition studies with L207R variant.** Time resolved fluorescence of association between 100 nM LOO10-GFP and 2.5 µM s10_short_ WT, with (blue curve) or without (red curve) pre-incubation with 10 µM s10_long_ L207R.
