# Supplementary material for: Substitutional landscape of a split fluorescent protein fragment using high-density peptide microarrays
Source: PLoS One. 2021 Feb 3;16(2):e0241461. doi: 10.1371/journal.pone.0241461 (PMC7857580; doi:10.1371/journal.pone.0241461)
Supplement: S10 Fig — Scatter plot comparing the median absolute deviation (MAD) across 12-replica for each variant before outlier removal and the standard deviation (SD) across 12-replica for the same variant after outlier removal. (DOCX) [file pone.0241461.s010.docx]

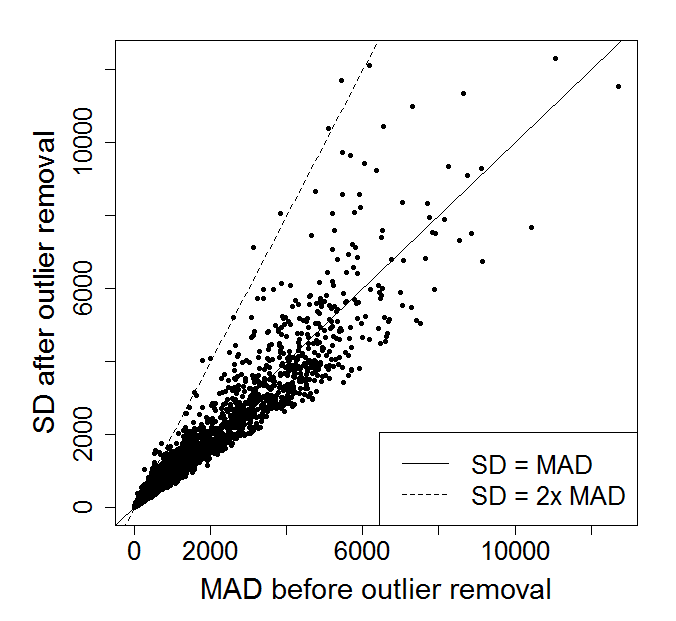


**S10 Fig. Quality control of the microarray data after outlier removal.** Scatter plot comparing the median absolute deviation (MAD) across 12-replica for each variant before outlier removal and the standard deviation (SD) across 12-replica for the same variant after outlier removal.
