# Supplementary material for: Substitutional landscape of a split fluorescent protein fragment using high-density peptide microarrays
Source: PLoS One. 2021 Feb 3;16(2):e0241461. doi: 10.1371/journal.pone.0241461 (PMC7857580; doi:10.1371/journal.pone.0241461)
Supplement: S11 Fig — Heatmap of mean fluorescence per sector, before normalization. Color key: Linear gradient between grey for dim sectors and green for bright sectors. (DOCX) [file pone.0241461.s011.docx]

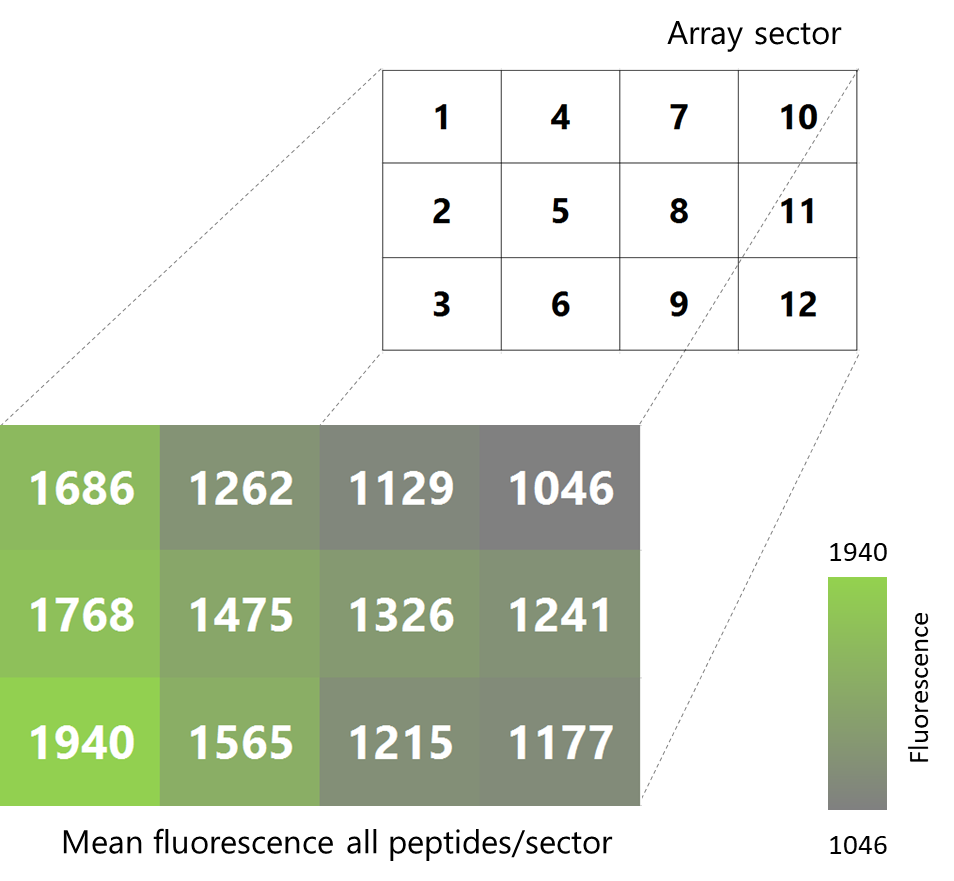


**S11 Fig. Fluorescence across the 12 microarray sectors.** Heatmap of mean fluorescence per sector, before normalization. Color key: Linear gradient between grey for dim sectors and green for bright sectors
