# Supplementary material for: Substitutional landscape of a split fluorescent protein fragment using high-density peptide microarrays
Source: PLoS One. 2021 Feb 3;16(2):e0241461. doi: 10.1371/journal.pone.0241461 (PMC7857580; doi:10.1371/journal.pone.0241461)
Supplement: S12 Fig — (A) Mean and (B) SD across the 12 replica for each variant before normalization plotted against the same quantity after normalization. (DOCX) [file pone.0241461.s012.docx]

*
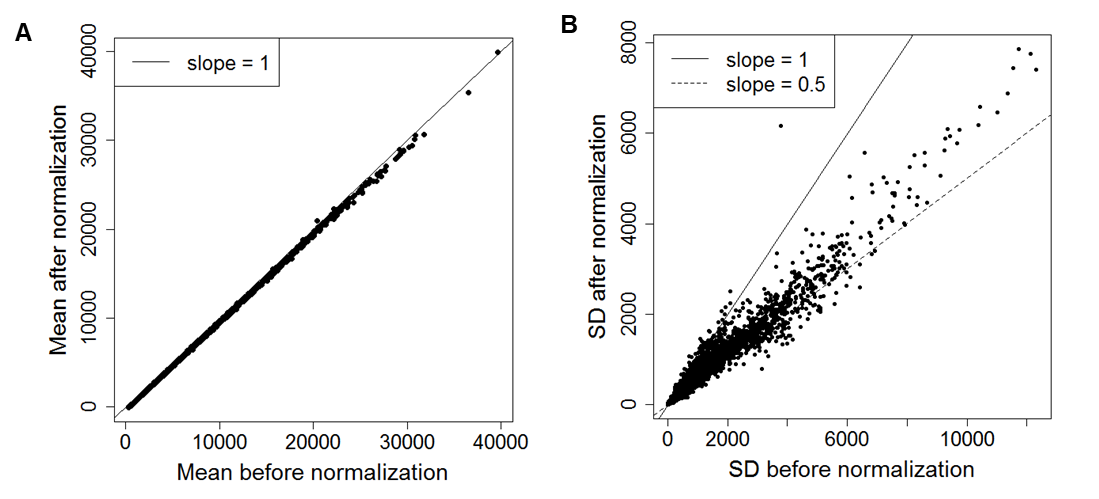
*

**S12 Fig. Quality control of the microarray data after cleaning.** (A) Mean and (B) SD across the 12 replica for each variant before normalization plotted against the same quantity after normalization.
