# Supplementary material for: Substitutional landscape of a split fluorescent protein fragment using high-density peptide microarrays
Source: PLoS One. 2021 Feb 3;16(2):e0241461. doi: 10.1371/journal.pone.0241461 (PMC7857580; doi:10.1371/journal.pone.0241461)
Supplement: S1 Table — Strand 10 substitutional variants and controls synthesized on the microarray. The linker sequences are gs2: GS; gs7: GSGSGSG, gk7: GKGSKSG and ge7: GEGSESG. (DOCX) [file pone.0241461.s013.docx]

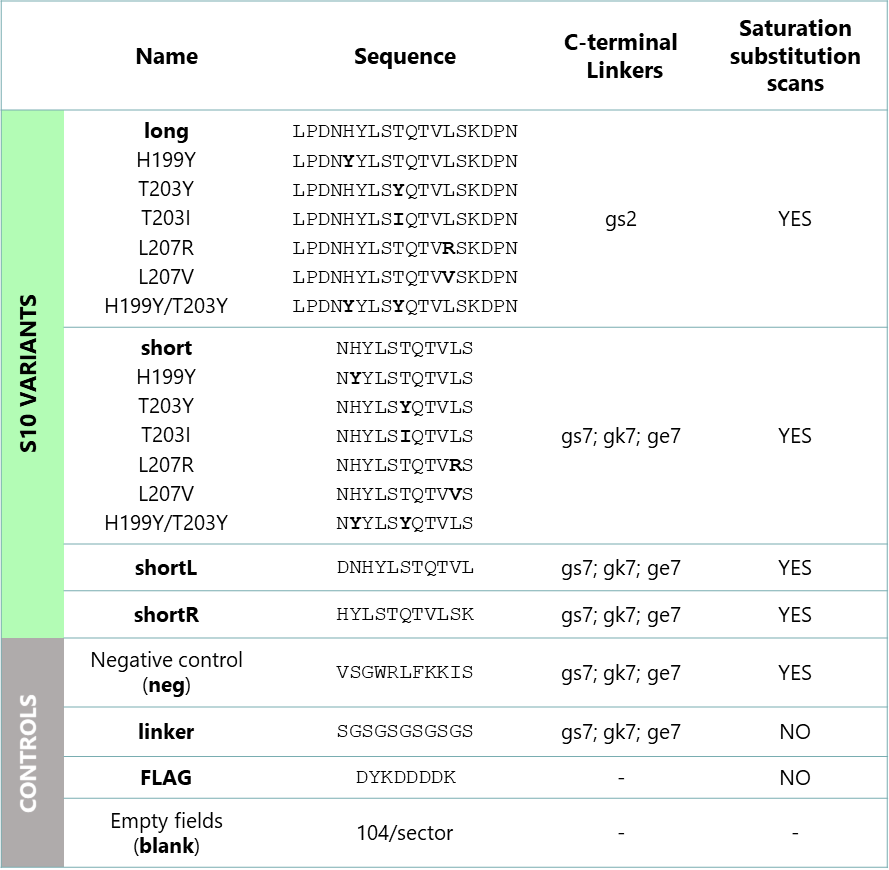


***S1 Table. Overview of the composition of the peptide library.***

*Strand 10 substitutional variants and controls synthesized on the microarray. The linker sequences are gs2: GS; gs7: GSGSGSG, gk7: GKGSKSG and ge7: GEGSESG.*
